# Characterization of the DYX2 locus on chromosome 6p22 with reading disability, language impairment, and IQ

**DOI:** 10.1007/s00439-014-1427-3

**Published:** 2014-02-09

**Authors:** John D. Eicher, Natalie R. Powers, Laura L. Miller, Kathryn L. Mueller, Sara Mascheretti, Cecilia Marino, Erik G. Willcutt, John C. DeFries, Richard K. Olson, Shelley D. Smith, Bruce F. Pennington, J. Bruce Tomblin, Susan M. Ring, Jeffrey R. Gruen

**Affiliations:** 1 Department of Genetics, Yale University School of Medicine, New Haven, CT USA; 2 Department of Pediatrics, Yale University School of Medicine, New Haven, CT USA; 3 MRC Integrative Epidemiology Unit, School of Social and Community Medicine, University of Bristol, Bristol, UK; 4 Hearing, Language and Literacy, Murdoch Childrens Institute, Melbourne, Australia; 5 Department of Communication Sciences and Disorders, The University of Iowa, Iowa City, IA USA; 6 Child Psychopathology Unit, Scientific Institute, IRCCS Eugenio Medea, Bosisio Parini, Lecco Italy; 7 Centre de Recherche de l’Institut Universitaire en Santé Mentale de Québec, Québec, Canada; 8 Department of Psychiatry and Neuroscience, Université Laval, Québec, Canada; 9 Institute for Behavioral Genetics, University of Colorado, Boulder, CO USA; 10 Department of Psychology and Neuroscience, University of Colorado, Boulder, CO USA; 11 Departments of Pediatrics and Developmental Neuroscience, University of Nebraska Medical Center, Omaha, NE USA; 12 Department of Psychology, University of Denver, Denver, CO USA; 13 Investigative Medicine Program, Yale Child Health Research Center, Yale University School of Medicine, New Haven, CT USA

## Abstract

Reading disability (RD) and language impairment (LI) are common neurodevelopmental disorders with moderately strong genetic components and lifelong implications. RD and LI are marked by unexpected difficulty acquiring and processing written and verbal language, respectively, despite adequate opportunity and instruction. RD and LI—and their associated deficits—are complex, multifactorial, and often comorbid. Genetic studies have repeatedly implicated the DYX2 locus, specifically the genes *DCDC2* and *KIAA0319*, in RD, with recent studies suggesting they also influence LI, verbal language, and cognition. Here, we characterize the relationship of the DYX2 locus with RD, LI, and IQ. To accomplish this, we developed a marker panel densely covering the 1.4 Mb DYX2 locus and assessed association with reading, language, and IQ measures in subjects from the Avon Longitudinal Study of Parents and Children. We then replicated associations in three independent, disorder-selected cohorts. As expected, there were associations with known RD risk genes *KIAA0319* and *DCDC2*. In addition, we implicated markers in or near other DYX2 genes, including *TDP2*, *ACOT13*, *C6orf62*, *FAM65B*, and *CMAHP*. However, the LD structure of the locus suggests that associations within *TDP2*, *ACOT13*, and *C6orf62* are capturing a previously reported risk variant in *KIAA0319*. Our results further substantiate the candidacy of *KIAA0319* and *DCDC2* as major effector genes in DYX2, while proposing *FAM65B* and *CMAHP* as new DYX2 candidate genes. Association of DYX2 with multiple neurobehavioral traits suggests risk variants have functional consequences affecting multiple neurological processes. Future studies should dissect these functional, possibly interactive relationships of DYX2 candidate genes.

**Electronic supplementary material:**

The online version of this article (doi:10.1007/s00439-014-1427-3) contains supplementary material, which is available to authorized users.

## Introduction

Communication disorders and learning disabilities are common, and can have long-lasting, adverse effects on affected individuals’ academic performance, self-esteem, and socioeconomic outcomes. Specifically, reading disability (RD or dyslexia) and language impairment (LI) affect 5–17 and 5–8 % of schoolchildren, respectively (Newbury et al. 2010; Pennington and Bishop 2009). RD and LI are characterized by unexpected difficulties with reading and verbal language, respectively, despite adequate educational and socioeconomic opportunity and instruction, as well as otherwise normal development (Pennington and Bishop 2009; Newbury et al. 2010). Written and verbal language are closely related and share several neurocognitive processes, including phonological processing and short-term memory (Catts et al. 2005; Gathercole and Baddeley 1990, 1993; Newbury et al. 2010; Pennington 2006; Pennington and Bishop 2009; Wise et al. 2007). In fact, children with LI are more likely to develop RD than their non-impaired peers (Pennington and Bishop 2009).

**Fig. 1:**
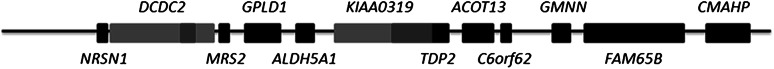
Schematic of the genes within the DYX2 locus on chromosome 6p22. Genes in *blue*, *DCDC2* and *KIAA0319*, have replicated associations with written and verbal language phenotypes, namely RD and LI. Regions in *red mark* two functional variants, READ1 in *DCDC2* and a risk haplotype containing markers in *KIAA0319* and *TDP2*, which have been functionally associated with RD and LI using animal models and molecular techniques

RD and LI are complex disorders with substantial genetic and environmental components (Bishop and Hayiou-Thomas 2008; Viding et al. 2004). For instance, the DYX2 locus on chromosome 6p22 has been consistently implicated in RD (Fig. 1) (Cardon et al. 1994; Deffenbacher et al. 2004; Gayán et al. 1999; Kaplan et al. 2002). Two DYX2 genes, *DCDC2* and *KIAA0319*, have been identified as RD risk genes, with considerable genetic and functional molecular evidence supporting the involvement of each (Couto et al. 2010; Cope et al. 2005; Elbert et al. 2011; Francks et al. 2004; Harold et al. 2006; Lind et al. 2010; Luciano et al. 2007; Marino et al. 2012; Meng et al. 2005; Newbury et al. 2011; Paracchini et al. 2006, 2008; Powers et al. 2013; Scerri et al. 2011; Schumacher et al. 2006; Wilcke et al. 2009; Zhong et al. 2013; Zou et al. 2012). Both *DCDC2* and *KIAA0319* function in neuronal migration during brain development, as demonstrated by in utero RNAi knockdown studies in rat (Meng et al. 2005; Paracchini et al. 2006). In both genes, putative functional variants appear to be regulatory in nature, as opposed to coding mutations. We previously identified a compound tandem repeat located within the breakpoints of a microdeletion in intron 2 of *DCDC2* as a risk variant (Marino et al. 2012; Meng et al. 2005, 2011; Powers et al. 2013). This repeat, known as regulatory element associated with dyslexia 1 (READ1), specifically binds the transcription factor ETV6 and appears capable of modulating expression from the *DCDC2* promoter (Meng et al. 2011; Powers et al. 2013). Within *KIAA0319*, the most strongly associated variant with RD is a 3-marker risk haplotype that spans approximately the 5′ half of the gene and some of its upstream sequence and neighboring gene *TDP2* (Cope et al. 2005; Francks et al. 2004; Paracchini et al. 2006). Both the risk haplotype itself and a putative functional SNP (rs9461045) in linkage disequilibrium with it have been shown to correlate with lower expression of the *KIAA0319* gene (Dennis et al. 2009; Elbert et al. 2011). Interestingly, there is evidence that READ1 and the *KIAA0319* risk haplotype interact in a non-additive fashion, suggesting that transcriptional co-regulation and interaction may play a key role in the relationship between the DYX2 locus and written/verbal language (Ludwig et al. 2008; Powers et al. 2013).

Although the literature suggests that variants in *DCDC2* and *KIAA0319* are predominantly responsible for the linkage and association signals from DYX2, other genes and elements may also contribute. The *KIAA0319* risk haplotype spans part of the neighboring gene *TDP2* (previously known as *TTRAP*), raising the question of whether the risk haplotype also tags risk variants in *TDP2* (Cope et al. 2005; Francks et al. 2004; Paracchini et al. 2006). *ACOT13* (previously known as *THEM2*), a gene adjacent to *TDP2*, was associated with asymmetry in functional activation of the superior temporal sulcus during reading tasks (Pinel et al. 2012). Genetic linkage analysis showed a linkage peak for full-scale and verbal IQ within *ALDH5A1*, located between *KIAA0319* and *DCDC2* (Plomin et al. 2004). Verbal IQ is correlated with reading and language skills, so this linkage result may reflect verbal skills measured during IQ testing. *ALDH5A1* encodes succinic semialdehyde dehydrogenase (SSADH), which influences the neuromodulator γ-hydroxybutyrate and the metabolism of γ-amino-butyric acid (GABA). Recent investigations have associated other neurotransmitter factors, including *DRD2*, *DRD4*, and *COMT*, with verbal language and LI, suggesting that neural signaling influences linguistic and cognitive traits (Beaver et al. 2010; Eicher et al. 2013a, b; Landi et al. 2013; Wong et al. 2013). Limited studies of other genes in the DYX2 locus suggest that they may also contribute to reading and language processes and their disorders.

The degree of relatedness of RD and LI indicates that they may share genetic and environmental risk factors. There are reports suggesting that some genes previously associated with RD also associate with LI, and vice versa. Within the DYX2 locus, *KIAA0319* has been associated with verbal language as well as reading (Newbury et al. 2011). The most widely studied gene in relation to language, *FOXP2*, was first implicated in verbal language disorders including dyspraxia of speech and LI, but various studies have expanded these associations with RD, as well as to endophenotypes identified using brain imaging methods (Fisher et al. 1998,; Kaminen et al. 2003; Lai et al. 2001; Peter et al. 2011; Pinel et al. 2012; Wilcke et al. 2011). Similarly, *CMIP* was first implicated in LI and later associated with reading-related traits (Newbury et al. 2009, 2011; Scerri et al. 2011). Recently, we performed a genome-wide association study (GWAS) on individuals with comorbid RD and LI, which identified *ZNF385D* as a contributor to processes underlying both disorders (Eicher et al. 2013b). There is strong evidence that RD and LI share genetic components; however, the specific genes and variants contributing to this shared genetic etiology remain largely unknown, as studies have typically been limited in overall number, number of genes examined, and statistical power. In particular, studies of the DYX2 locus have generally not covered the entire DYX2 locus or have tagged it incompletely, and have likely been underpowered to detect single-variant effects.

Therefore, the overall goal of this study was to characterize the associations of the DYX2 locus with RD, LI, and cognition as measured by IQ scores. To accomplish this task, we developed a single-nucleotide polymorphism (SNP) marker panel designed to capture the known common variation of the entire DYX2 locus. We genotyped this DYX2 marker panel in the Avon Longitudinal Study of Parents and Children (ALSPAC) and performed association analysis using reading, language, and cognitive measures collected between the ages of 7 and 9 years. We then replicated our associations in three cohorts selected for either RD or LI. We hypothesized that we would confirm the associations of *DCDC2* and *KIAA0319* with RD and LI and refine the locations of and possibly identify the variants responsible for these associations. Our results implicating a six-marker haplotype block in linkage disequilibrium with the READ1 element in *DCDC2* with Severe RD, and its non-additive genetic interaction with *KIAA0319* on reading, verbal language, and IQ performance are presented elsewhere (Powers et al. 2013). Here, we report the additional results of the DYX2 association scan of RD, LI, and IQ.

## Methods

### Subjects

Our discovery cohort in this investigation was the Avon Longitudinal Study of Parents and Children (ALSPAC). The ALSPAC is a population-based birth cohort based in Avon, United Kingdom. Subjects were recruited before birth—a total of 15,458 fetuses were recruited, of whom 14,701 were alive at 1 year of age. Recruitment, participants, and study methodologies are described in detail elsewhere (http://www.bristol.ac.uk/alspac) (Boyd et al. 2012; Golding et al. 2001). The study website contains details of all the data that are available through a fully searchable data dictionary (http://www.bris.ac.uk/alspac/researchers/data-access/data-dictionary). DNA samples for genetic analysis were available for 10,259 subjects. Reading, language, and IQ were assessed at ages 7, 8, and 9 years using standardized measures. We excluded subjects with IQ <75 on the Wechsler Intelligence Scale for Children (WISC-III) full scale IQ to prevent confounding effects of intellectual disability (Eicher et al. 2013a, b; Powers et al. 2013; Wechsler et al. 1992). To prevent population stratification in genetic analyses, we excluded subjects of non-European descent. Samples with overall genotype call rates <0.85 were also excluded from analyses. This resulted in a final sample of 5,579 individuals for language-related analyses and 5,525 individuals for reading-related analyses. Ethical approval was obtained from the ALSPAC Ethics and Law Committee, Local UK Research Ethics Committees, and the Yale Human Investigation Committee.

Following discovery analyses in the ALSPAC, we replicated associated markers in three cohorts specifically recruited for either RD or LI (Table 1). The Iowa LI cohort is composed of 219 LI cases and 209 sex- and age-matched, unrelated controls collected at the University of Iowa. Subjects within the Iowa LI cohort completed a battery of language measures, which were used to derive a composite language score. This composite score was then dichotomized into case–control status at −1.14 standard deviations (Eicher et al. 2013a; Tomblin et al. 1996; Weismer et al. 2000). The Colorado Learning Disabilities Research Center (CLDRC) or Colorado RD cohort consists of 1,188 individuals within 292 families of twin pairs and their siblings. Families were recruited to the study if at least one member of each twin pair had a history of reading problems (Meng et al. 2005). Within the Colorado RD cohort, RD cases were defined as individuals with a discriminant score below the mean of an age- and sex-matched sample of twins with no school history for RD. The discriminant score is a weighted composite of the reading recognition, comprehension, and spelling subtests of the Peabody Individual Achievement test (PIAT) (Gayán et al. 1999). In the case of monozygotic twins, only one member of each twin pair was used for this study. The Italian cohort consists of 878 individuals in 304 nuclear families; these families were recruited via a proband with clinically diagnosed RD (Marino et al. 2012). Probands were diagnosed with RD if they scored two or more standard deviations below expected grade level on speed or accuracy on text, word, or nonword reading, had a full-scale IQ of at least 85, and did not have any sensory or neurological disorder (Marino et al. 2012). Ethical approval for recruitment and study methodologies were obtained from the Yale Human Investigation Committee, Institutional Review Boards at the University of Iowa, the University of Denver, University of Colorado-Boulder, University of Nebraska Medical Center, and the Scientific Review Board and the Ethical Committee of the Eugenio Medea Scientific Institute.

**Table 1:** Replication cohorts

	Iowa LI	Colorado RD	Italy RD
Cohort-type	Case–control	Family-based	Family-based
Number of subjects	428	1,188	878
Number of families	N/A	292	304
Disorder	LI	RD	RD
Analysis	SVS	TDT (PLINK)	TDT (PLINK)
Association conditioned on:	Case–control status	Case–control status, discriminant score	Case–control status
Case status determined by:	Composite score on language measures	Composite discriminant score on reading tasks	Text-, single-word, or non-word reading tasks

### ALSPAC reading, language, and IQ measures

In ALSPAC, reading measures used in this investigation included a phoneme deletion task at age 7 years, single-word reading tasks at ages 7 and 9 years, and a single non-word reading task at age 9 years (Table 2). The phoneme deletion task, also known as the Auditory Analysis test, measures phoneme awareness, a core deficit in RD (Rosner and Simon 1971). For this task, the child listens to a word spoken aloud and is then asked to remove a specific phoneme from that word to make a new word. Single-word reading was assessed at age 7 years using the reading subtest of the Wechsler objective reading dimensions (WORD) (Rust et al. 1993). At age 9 years, single-word reading was again assessed by asking the child to read ten real words and ten non-words aloud (Nunes et al. 2003). To examine severe cases (Severe RD), we defined cases as having a score 2 or more standard deviations below the mean on the phoneme deletion task (Table 3). We also defined cases with Moderate RD as scoring at least 1 standard deviation below the mean on single-word reading at age 7 years, single-word reading at age 9 years, and single non-word reading at age 9 years (Table 3). We chose a threshold of 1 standard deviation as we included three different measures to isolate individuals with persistently poor decoding skills. We examined different severity levels because past studies in the DYX2 locus have shown differences in genetic association patterns depending on case severity, particularly with *KIAA0319* associating with more moderate RD case definitions and *DCDC2* with more severe definitions (Paracchini et al. 2008; Powers et al. 2013; Scerri et al. 2011).

**Table 2:** ALSPAC phenotype measures

Measure	Domain
Phoneme deletion (PD) age 7 years	Reading
Single-word reading (SWR7) age 7 years	Reading
Single non-word reading (SNR) age 7 years	Reading
Single-word reading (SWR9) age 9 years	Reading
Wechsler objective language dimensions (WOLD) verbal comprehension age 8 years	Language
Nonword repetition task (NWR) age 8 years	Language
Wechsler Intelligence Scale for Children (WISC) full scale IQ (FSIQ) age 8 Years	IQ
Wechsler Intelligence Scale for Children (WISC) verbal IQ (VIQ) age 8 Years	IQ
Wechsler Intelligence Scale for Children (WISC) performance IQ (PIQ) age 8 Years	IQ

**Table 3:** Phenotype definitions for ALSPAC analyses

	Phenotype definition
Reading (RD)	
Severe RD	2 Standard deviations below sample mean on the phoneme deletion task
Moderate RD	1 Standard deviation below sample mean on SWR7, SNR, and SWR tasks
Language (LI)	
Severe LI	2 Standard deviations below sample mean on either WOLD and/or NWR tasks
Moderate WOLD	1.5 Standard deviations below sample mean on the WOLD task
Moderate NWR	1.5 Standard deviations below sample mean on the NWR task
Cognition (IQ)	
Total IQ	Quantitative performance on WISC Total IQ task
Verbal IQ	Quantitative performance on WISC Verbal IQ task
Performance IQ	Quantitative performance on WISC Performance IQ task

Language measures were collected at age 8 years (Table 2). An adaptation of the nonword repetition task (NWR), in which subjects repeated recordings of nonwords, was used to assess short-term phonological memory and processing abilities (Gathercole and Baddeley 1996). Children also completed the Wechsler objective language dimensions (WOLD) verbal comprehension task at age 8 years (Wechsler 1996), where they answered questions about a paragraph read aloud by an examiner describing a presented picture. We chose these measures because individuals with LI are known to perform consistently poorly on NWR and WOLD tasks (Bishop et al. 1996; Newbury et al. 2009). As with RD, we were interested in the association of the DYX2 locus in relation to severity of LI. Here, we defined severe LI cases by scores of 2 or more standard deviations below the sample mean on either language task (severe LI) (Table 3). In contrast, we defined two classes of moderate cases as scoring at least 1.5 standard deviations below the sample mean on either the NWR or WOLD task each task (moderate NWR and moderate WOLD) (Table 3). Verbal IQ, performance IQ, and full scale IQ were assessed at age 8 years, using the Wechsler Intelligence Scale for Children (WISC-III) (Table 2). IQ measures were examined as quantitative traits (Table 2).

### Genotyping and genetic analyses

We developed a SNP marker panel in an attempt to capture the common variation in the DYX2 locus. TagSNPs in the locus were selected using the association study design server of Han et al. (2008). The final DYX2 panel contained 195 SNPs with an estimated average power of 83 and 68 % to capture known common and rare variants, respectively, in the DYX2 locus spanning approximately 1.4 Mb. Markers were genotyped on the Sequenom MassARRAY platform (San Diego, CA) following manufacturer’s guidelines at the Yale Center for Genome Analysis (West Haven, CT). Briefly, markers were genotyped in nine multiplex reactions of 30–36 markers each, totaling 300 markers (Supplemental Table 1). A subset of markers was not in the DYX2 locus and was not included in the subsequent characterization of the DYX2 locus. In addition to quality control via call rate and Hardy–Weinberg, the histogram plot for each marker was manually evaluated, and a total of 15 markers showing aberrant patterns were excluded. To control for errors in labeling and manipulation of plates, the samples were also genotyped for four sex-determining SNPs in the genes *AMELXY* and *ZNFXY* in the pseudoautosomal regions of the X and Y chromosomes. These SNPs correctly determined sex for 99.5 % of samples; the remaining samples were excluded.

Markers that deviated substantially from Hardy–Weinberg equilibrium (*p* < 0.0001), or that had an overall call rate <85 %, were not used for genetic analyses. In the discovery ALSPAC cohort, single marker SNP analyses of case–control status and quantitative traits were completed using SNP and variation suite (SVS) v7.6.4 (Bozeman, MT). Linkage disequilibrium was assessed and haplotype blocks were constructed using the four-gamete rule option in HaploView v4.2. Haplotype-based association tests were performed with haplotypes that had frequencies ≥1 % using PLINK v1.07 (Barrett et al. 2005; Purcell et al. 2007). To correct for multiple testing, we used a Bonferroni threshold of 0.000256 (0.05 divided by 195 markers) for discovery association tests in the ALSPAC cohort. However, associations with *p* < 0.001 are also reported for the ALSPAC discovery cohort to indicate suggestive results.

We tested SNPs that had single marker or within-haplotype associations with *p* < 0.001 in the ALSPAC for replication in the Iowa LI, Italian RD, and Colorado RD cohorts. Iowa LI was analyzed using SVS v7.6.4 (Bozeman, MT), while the family-based Italian RD and Colorado RD cohorts were examined using PLINK v1.07 (Purcell et al. 2007). We moved suggestive ALSPAC results forward to our replication analyses to emphasize replication of associations over statistical corrections for multiple testing. Replications with* p* < 0.05 in the Iowa LI, Italian RD, and Colorado RD cohorts are reported.

## Results

We performed association with DYX2 markers in three separate domains: (1) RD, (2) LI, and (3) IQ. For the sake of clarity, we present our association findings domain-by-domain, with an emphasis on replication as opposed to correction for multiple testing.

### RD

We performed associations with RD using two different severity definitions: (1) Severe RD and (2) Moderate RD (Table 3). For Severe RD, we observed single marker associations with *KIAA0319* and *TDP2* (Table 4). There was an association of a six-marker haplotype within *DCDC2* that is linked to the risk element READ1 and Severe RD that is explored fully in Powers et al. (2013). *TDP2* marker rs2294691 did not replicate its association in any of the three replication cohorts (Table 6). However, *KIAA0319* marker rs10456309 did replicate in Iowa LI and Colorado RD cohorts (Table 6). With Moderate RD, there was an association between rs1562422 near the gene *FAM65B* and the pseudogene *CMAHP*, which was replicated in the Colorado RD cohort (Tables 4, 6).

**Table 4:** Single marker genetic associations with various RD and LI case–control definitions

Phenotype	Marker	Gene	BP location	Model	OR (95 % CI)	*p* value
Severe RD	rs2294691	*TDP2*	24,652,843	Allelic	2.0 (1.3–2.9)	0.00050
Severe RD	rs2294691	*TDP2*	24,652,843	Additive	1.9 (1.3–2.8)	0.00053
Severe RD	rs2294691	*TDP2*	24,652,843	Dominant	2.3 (1.5–3.7)	0.00018*
Severe RD	rs10456309	*KIAA0319*	24,589,562	Recessive	10.5 (2.2–49.5)	0.00020*
Moderate RD	rs1562422	*CMAHP*	25,044,577	Dominant	1.7 (1.2–2.2)	0.00081
Severe LI	rs807694	*DCDC2*	24,303,383	Additive	1.8 (1.3–2.5)	0.00057
Severe LI	rs807694	*DCDC2*	24,303,383	Allelic	1.8 (1.3–2.5)	0.00050
Severe LI	rs807694	*DCDC2*	24,303,383	Dominant	1.9 (1.3–2.7)	0.00062
Moderate WOLD	rs3756814	*C6orf62*	24,705,835	Additive	0.7 (0.6–0.9)	0.00039
Moderate WOLD	rs3756814	*C6orf62*	24,705,835	Allelic	0.7 (0.6–0.9)	0.00047
Moderate WOLD	rs3777663	*ACOT13*	24,700,235	Additive	0.6 (0.5–0.8)	0.00039
Moderate WOLD	rs3777663	*ACOT13*	24,700,235	Allelic	0.7 (0.5–0.8)	0.00041

* Genetic association survives correction for multiple testing

### LI

Association tests were performed on three LI phenotypes: (1) severe LI, (2) moderate NWR, and (3) moderate WOLD (Table 3). As with severe RD, there were associations between *DCDC2* and Severe LI. The *DCDC2* haplotype that associated with severe LI is discussed in Powers et al. (2013). A marker within this *DCDC2* haplotype, rs807694, showed association with Severe LI and was replicated in the Iowa LI cohort (Tables 4, 6). With a more moderate case definition, we observed associations with *ACOT13* and *C6orf62* (Table 4), genes neighboring *KIAA0319* and *TDP2*. Both rs3777663 in *ACOT13* and rs3756814 in *C6orf62* showed associations in the Italian RD and Iowa LI cohorts (Table 6).

### IQ

We also performed association tests between DYX2 markers and verbal IQ, performance IQ, and full scale IQ (Table 2). Verbal IQ associations included single markers and haplotypes covering the 5′ half of *KIAA0319*, rs9348646 in *FAM65B*, and a haplotype spanning *ACOT13* and *C6orf62*, with evidence of replication (Tables 5a, b, 6). There was substantial overlap of DYX2 associations with verbal IQ and associations with RD and LI. This finding may be a result of the high correlations among these traits (Table 7). The associations of DYX2 with performance IQ and full scale IQ were weaker; there were no associations with performance IQ and a single, non-replicated association of full scale IQ with rs2328791, which is located in a large intergenic region telomeric to *NRSN1* and *DCDC2* (Tables 5a, b, 6).

**Table 5:** Single marker (a) and haplotype-based (b) genetic associations with quantitative measure of cognition

(a) Single marker genetic associations with cognition						
Phenotype	Marker	Gene	BP location	Model	Slope	*p* value
Verbal IQ	rs9295626	*KIAA0319*	24,587,339	Allelic	1.40	0.00041
Verbal IQ	rs9295626	*KIAA0319*	24,587,339	Additive	1.39	0.00043
Verbal IQ	rs7763790	*KIAA0319*	24,615,063	Allelic	−1.40	0.00045
Verbal IQ	rs7763790	*KIAA0319*	24,615,063	Additive	−1.38	0.00048
Verbal IQ	rs6935076	*KIAA0319*	24,644,322	Allelic	1.16	0.00049
Verbal IQ	rs6935076	*KIAA0319*	24,644,322	Additive	1.15	0.00052
Verbal IQ	rs9348646	*FAM65B*	24,052,526	Allelic	−1.14	0.00066
Verbal IQ	rs9348646	*FAM65B*	24,052,526	Additive	−1.14	0.00066
Full scale IQ	rs2328791	N/A	23,736,848	Allelic	−1.21	0.00066
Full scale IQ	rs2328791	N/A	23,736,848	Additive	−1.18	0.00075
Full scale IQ	rs2328791	N/A	23,736,848	Recessive	−3.36	0.00042

(b) Haplotype-based genetic associations with cognition					
Markers	Haplotype	Gene	BP location	Slope	*p* value
rs2817201, rs9295626	AT	*KIAA0319*	24,585,214, 24,587,339	1.42	0.000378
rs10456309, rs4576240, rs17307478, rs9356939, rs7763790, rs6456621	GGTCAC	*KIAA0319*	24,589,562, 24,596,478, 24,605,024 24,613,354, 24,615,063, 24,618,511	−1.40	0.000569
rs6456624, rs6935076, rs2038137, rs3756821, rs1883593, rs3212236	AGATA	*KIAA0319*	24,639,223, 24,644,322, 24,645,943, 24,646,821, 24,647,191, 24,648,455	1.81	0.0000145*
rs3777663, rs3756814, rs6931809, rs6916186, rs6933328, rs17491647	TGTGGA	*ACOT13/C6orf62*	24,700,235, 24,705,835, 24,706,770, 24,708,523, 24,710,920, 24,713,723	−1.56	0.000742

* Genetic association survives correction for multiple testing

**Table 6:** Replication of genetic associations in the Iowa, Italian, and Colorado cohorts

Marker	Gene	Iowa case control		Italy case control		Colorado case control		Colorado discriminant score	
		OR	*p*	OR	*p*	OR	*p*	Slope	*p*
rs2328791	N/A	1.0	0.813	1.0	1.000	0.9	0.646	0.087	0.447
rs33914824^a^	*DCDC2*	**2.2**	**0.034**	0.9	0.768	1.1	0.847	0.023	0.934
rs807694^a^	*DCDC2*	**1.9**	**0.028**	0.9	0.786	0.9	0.853	−0.025	0.919
rs707864^a^	*DCDC2*	**1.6**	**0.017**	1.0	0.840	1.2	0.446	−0.246	0.101
rs10456301^a^	*DCDC2*	0.9	0.553	1.1	0.811	1.5	0.289	0.221	0.162
rs16889066^a^	*DCDC2*	1.2	0.517	1.0	0.884	1.2	0.622	−0.304	0.150
rs9379651^a^	*DCDC2*	1.1	0.602	1.3	0.225	0.6	0.059	0.205	0.141
rs2817201	*KIAA0319*	1.1	0.733	1.2	0.129	1.0	1.000	0.034	0.787
rs9295626	*KIAA0319*	1.1	0.579	**0.6**	**0.0055**	1.0	0.823	−0.158	0.169
rs10456309	*KIAA0319*	**0.5**	**0.073**	0.7	0.189	0.4	0.206	**0.628**	**0.0133**
rs4576240	*KIAA0319*	1.1	0.825	**1.9**	**0.0027**	1.1	0.862	−0.052	0.754
rs17307478	*KIAA0319*	1.0	0.996	1.3	0.292	0.8	0.555	0.039	0.803
rs9356939	*KIAA0319*	**4.0**	**0.018**	0.8	0.069	1.3	0.151	−0.116	0.254
rs7763790	*KIAA0319*	1.0	0.831	1.1	0.627	1.4	0.163	0.014	0.910
rs6456621	*KIAA0319*	**2.2**	**0.019**	1.6	0.405	1.8	0.366	−0.458	0.104
rs3756821	*KIAA0319*	1.2	0.278	1.0	0.842	1.2	0.327	−0.033	0.734
rs1883593	*KIAA0319*	1.3	0.169	**1.6**	**0.0052**	1.3	0.239	−0.108	0.395
rs3212236	*KIAA0319*	1.0	0.883	1.1	0.496	0.9	0.745	−0.124	0.319
rs2294691	*TDP2*	1.1	0.779	1.9	0.0578	1.4	0.491	−0.290	0.247
rs3777663	*ACOT13*	**0.7**	**0.016**	**0.6**	**0.0052**	1.0	0.908	0.101	0.345
rs3756814	*C6orf62*	**0.7**	**0.005**	**0.7**	**0.023**	0.9	0.600	−0.003	0.980
rs6931809	*C6orf62*	**1.4**	**0.023**	**1.4**	**0.017**	1.2	0.491	−0.096	0.382
rs6916186	*C6orf62*	0.9	0.757	1.2	0.413	1.2	0.547	0.112	0.490
rs6933328	*C6orf62*	0.9	0.612	0.9	0.613	1.0	0.827	**0.215**	**0.0515**
rs17491647	*C6orf62*	0.8	0.155	0.7	0.104	1.0	0.901	0.042	0.709
rs9348646	*FAM65B*	0.9	0.358	1.1	0.535	1.4	0.144	**−0.415**	**0.00051**
rs1562422	*CMAHP*	1.0	0.793	1.0	0.796	**0.6**	**0.093**	−0.030	0.840

^a^These markers are part of the six-marker risk haplotype in *DCDC2* fully discussed in Powers et al. (2013)

Bold denotes nominally associated markers

**Table 7:** Phenotype correlations in the ALSPAC cohort

	NWR	WOLD	SWR7	SWR9	SNR	PD	FSIQ	VIQ	PIQ
NWR	1								
WOLD	0.214	1							
SWR7	0.403	0.259	1						
SWR9	0.351	0.202	0.722	1					
SNR	0.306	0.149	0.660	0.708	1				
PD	0.362	0.165	0.688	0.550	0.538	1			
FSIQ	0.324	0.386	0.500	0.387	0.343	0.406	1		
VIQ	0.346	0.424	0.536	0.421	0.421	0.426	0.871	1	
PIQ	0.192	0.216	0.292	0.218	0.218	0.246	0.819	0.435	1

All correlations *p* < 0.05

*NWR* nonword repetition age 8 years, *WOLD* Wechsler objective language dimensions verbal comprehension age 8 years, *SWR7* single word reading age 7 years, *SWR9* single word reading age 9 years, *SNR* single nonword reading age 9 years, *PD* phoneme deletion age 7 years, *FSIQ* full scale IQ age 8 years, *VIQ* verbal IQ age 8 years, *PIQ* performance IQ age 8 years

### Linkage disequilibrium within DYX2

**Fig. 2:**
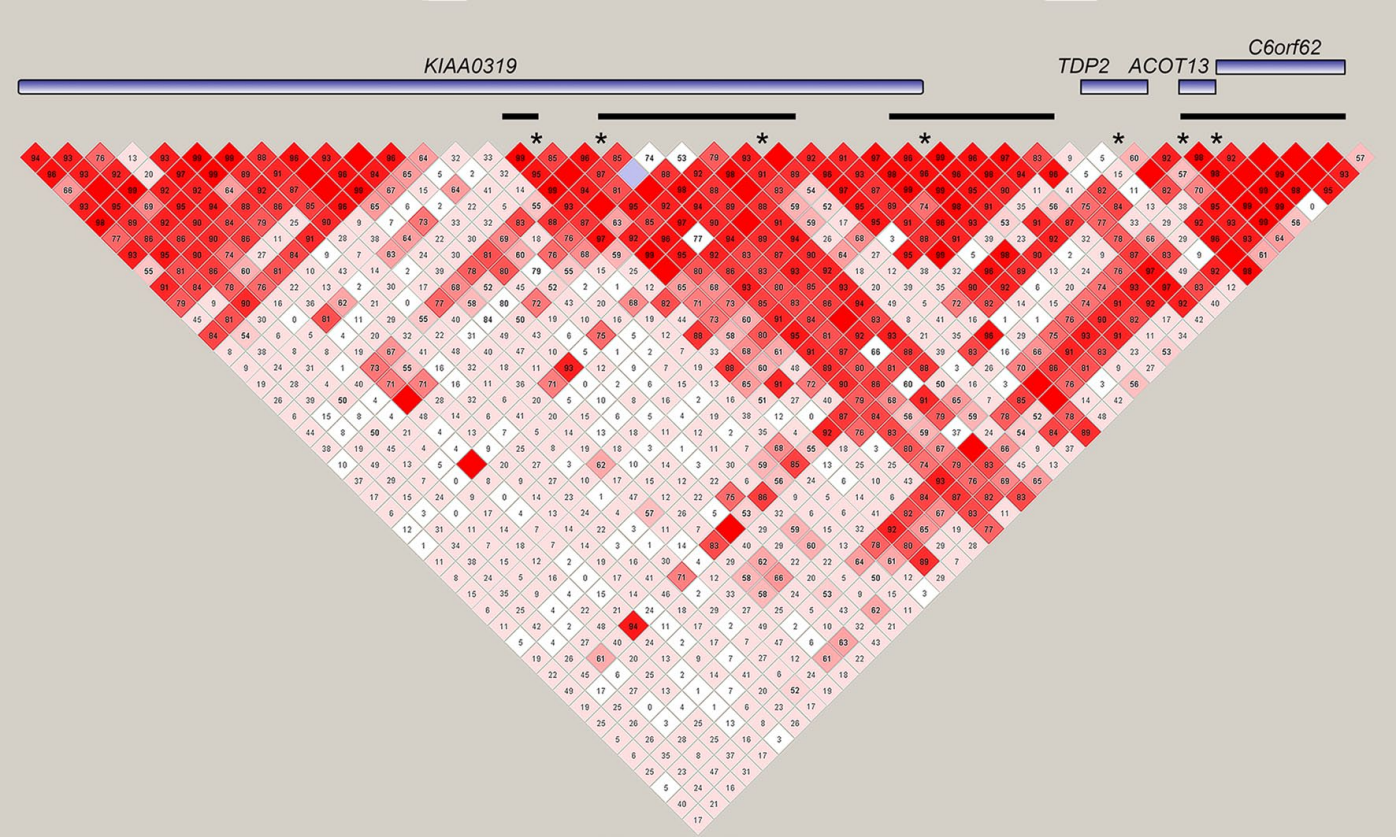
Linkage disequilibrium between associated markers and haplotypes in *KIAA0319*, *TDP2*, *ACOT13*, and *C6Orf62* for RD, LI, and/or verbal IQ. *Asterisks* represent single-marker associations, while *black bars* represent associations with haplotypes. *Numbered boxes* show LD (as measured by D’) between markers. Genes are not drawn to scale; the size of a gene in the diagram depends on the number of markers in our panel that localize to that gene

**Fig. 3:**
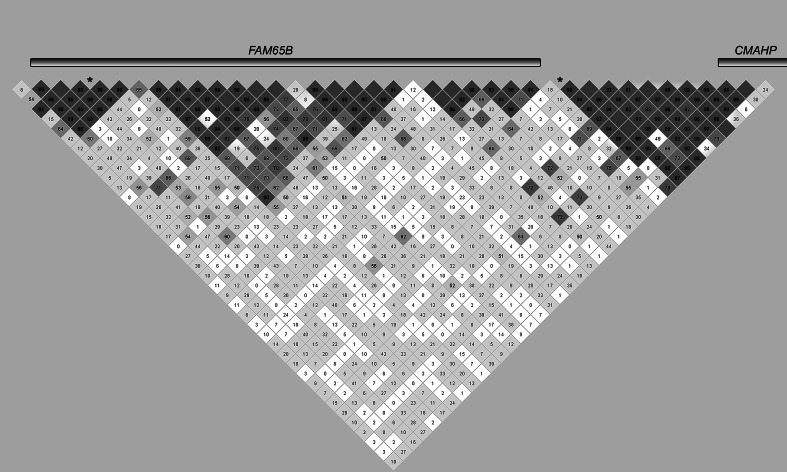
Linkage disequilibrium between associated markers and haplotypes in *FAM65B* and *CMAHP* for RD or verbal IQ. *Asterisks* represent single-marker associations. *Numbered boxes* show LD (as measured by D′) between markers. Genes are not drawn to scale; the size of a gene in the diagram depends on the number of markers in our panel that localize to that gene

In our analyses, we observed replicated associations in the following genes: *DCDC2*, *KIAA0319*, *TDP2*, *ACOT13*, *C6orf62*, *FAM65B*, and the pseudogene *CMAHP*. However, as these SNPs are in close proximity to each other, we next assessed linkage disequilibrium (LD) among our marker panel to determine whether the associated SNPs were tagging the same variation in the locus. Our previous work showed that *DCDC2* associations tagged READ1 alleles (Powers et al. 2013). Within *KIAA0319*, there appears to be two clear LD blocks separating the gene into a 5′ half and a 3′ half (Fig. 2). The 5′ half of *KIAA0319* is in strong LD with *TDP2*, *ACOT13*, and *C6orf62*, indicating that associations within these genes may be capturing that same variation (Fig. 2). Associations in *FAM65B* and *CMAHP* appear to be tagging independent associations (Fig. 3). Although rs1562422 is located intergenic to *FAM65B* and *CMAHP*, this marker is in strong LD with other markers within the *CMAHP* pseudogene. Integration of our association analyses and LD structure indicate four independent association signals centered on (1) *DCDC2*, (2) the 5′ half of *KIAA0319*, (3) *FAM65B*, and (4) *CMAHP*.

## Discussion

**Fig. 4:**
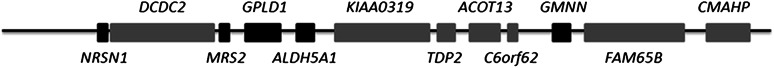
An updated schematic of genes in our study with markers that show replicated associations with RD, LI, and/or IQ. The list of these genes (shown in *blue*) has expanded to seven (*DCDC2*, *KIAA0319*, *TDP2*, *ACOT13*, *C6orf62*, *FAM65B*, and *CMAHP*), although linkage disequilibrium may account for multiple associations (particularly for *KIAA0319*, *TDP2*, *ACOT13*, and *C6orf62*)

In this investigation, we characterized the relationship of the DYX2 locus with RD, LI, and IQ (Fig. 4). Our results confirm the associations of RD risk genes *KIAA0319* and *DCDC2* with LI. Additionally, we identify *FAM65B* and *CMAHP* as candidate genes for linguistic traits. Markers within the DYX2 locus showed association with multiple aspects of communication, including RD, LI, and verbal IQ. However, there was a marked absence of DYX2 associations with full scale IQ and performance IQ, suggesting that the DYX2 locus influences language-related processes to a greater extent than general cognition.

The genetic association of DYX2 with RD, LI, and verbal IQ is the latest example of various neurocognitive and communication processes sharing genetic associations. Our group and others have shown that these neurobehavioral traits have common genetic contributors, including variants in *FOXP2*, *KIAA0319*, *CMIP*, *ZNF385D*, *CNTNAP2*, and *DCDC2* (Eicher et al. 2013b; Newbury et al. 2009, 2011, Pennington and Bishop 2009, Peter et al. 2011; Pinel et al. 2012; Powers et al. 2013; Scerri et al. 2011; Wilcke et al. 2011). The expansion of DYX2’s association from reading to include other language-related processes suggests that the causative variants may affect reading and verbal language in a pleiotropic manner, as opposed to one or the other exclusively. Our findings provide additional evidence for a ‘generalist genes hypothesis,’ which is also supported by a recent genome-wide complex trait analysis (GTCA) of cognitive and learning abilities (Trzaskowski et al. 2013). The strong correlations and relatedness among these neurocognitive measures (Table 7) suggest that these DYX2 genes affect neurocognitive processes central to language learning, which in turn manifest themselves phenotypically in various ways, including reading, language, and cognition.

That multiple DYX2 genes showed association with the phenotypes in this study is interesting, and at first glance somewhat unexpected. One possibility is that one or two genes are not solely responsible for the consistent implication of this locus in reading, language, and cognitive phenotypes. *KIAA0319* and *DCDC2* are currently considered as the two major risk genes in the DYX2 locus. Both genes have been implicated in both RD and sub-clinical variation in reading performance, using both classical neurobehavioral measures, and more recently, neuroimaging techniques (Eicher and Gruen 2013; Graham and Fisher 2013). Other genes in DYX2 have been associated with RD, although not nearly as often as *DCDC2* and *KIAA0319*. In this study, with a dense SNP panel, we were able to observe associations with other DYX2 elements, including *FAM65*B and *CMAHP*. It would appear possible that instead of a single gene—or in this case two genes (*DCDC2* and *KIAA0319*)—multiple elements, possibly regulatory in nature, within DYX2, influence language processes. However, DYX2 has repeatedly shown strong linkage to RD, a pattern generally indicative of one or two variants of large effect, and not of multiple independent variants with more modest effect sizes.

Another possible explanation for the number of DYX2 genes associating in this study is LD within the DYX2 locus. In fact, LD likely explains the cluster of associations around *KIAA0319*, *TDP2*, *ACOT13*, and *C6orf62*. As shown in Fig. 2, two major LD blocks span *KIAA0319*—one spans the 3′ half of the gene, while the other spans the 5′ region of *KIAA0319* as well as *ACOT13*, *TDP2*, and part of *C6orf62*. Nearly all of the associations in this study localize to this 5′ LD block, which also contains the previously reported *KIAA0319* RD risk haplotype (Cope et al. 2005; Francks et al. 2004; Paracchini et al. 2006). Because of this LD structure, it is impossible to determine whether the associations in this region are independent or are capturing the same functional variant. We consider the latter possibility the most likely and believe that the associations in this region are likely tagging the same causative variant captured by the *KIAA0319* RD risk haplotype. Functional study of this region—particularly of the less studied genes *TDP2*, *ACOT13*, and *C6orf62*—will almost certainly be necessary to determine whether these associations are independent or not.

By contrast, the markers within or near *FAM65B* and *CMAHP* appear to be capturing distinct association signals from two different LD blocks (Fig. 3). The SNP rs9348646, which showed association with verbal IQ, is located within an intron of *FAM65B* in one LD block, while rs1562422, which showed association with moderate RD, localized to a separate LD block. While rs1562422 is an intergenic marker located physically between *FAM65B* and *CMAHP*, it shows strong LD with markers in *CMAHP* (Fig. 3). The LD patterns within the DYX2 locus suggest that associations in *KIAA0319*, *TDP2*, *ACOT13*, and *C6orf62* are tagging the same causative variant, while rs9348646 in *FAM65B* and rs1562422 near *CMAHP* are independent.

The other DYX2 genes, including *FAM65B* and *CMAHP*, have been less studied than the established risk genes *DCDC2* and *KIAA0319*. Little is known about *FAM65B* in terms of biological function; however, there is evidence that *FAM65B* may influence migration in T lymphocytes (Rougerie et al. 2013). Animal models of *DCDC2* and *KIAA0319* have implicated these genes in migratory processes, albeit in a neural context. *CMAHP*, which encodes a key enzyme in the synthesis of the sialic acids Neu5Ac and Neu5Gc in other mammals, was rendered a pseudogene in humans by an inactivating microdeletion and subsequent fixation of the inactive allele in early human populations (Chou et al. 1998). Although *ACOT13* appears to be tagging variation within *KIAA0319*, the preliminary functional studies of *ACOT13* are intriguing. *ACOT13* was recently associated with lower asymmetric activation of the posterior superior temporal sulcus during reading and phonology tasks (Pinel et al. 2012). The protein product encoded by *ACOT13* has been co-localized with beta-tubulin on microtubules; microtubule binding is postulated to be important to RD, as *DCDC2* contains two doublecortin domains that are thought to bind microtubules (Cheng et al. 2006).

Genes and regulatory elements within the DYX2 locus may contribute interactively to reading and language domains, as seen with the apparent non-additive relationship between putative regulatory variants in *DCDC2* and *KIAA0319* (Powers et al. 2013, Ludwig et al. 2008). These risk variants have been shown to influence gene expression and to interact with each other to substantially influence performance on reading- and language-related tasks. It is likely that a complex network, where regulatory elements interact and co-regulate other DYX2 genes and elements, contributes to reading, language, and cognitive phenotypes. If so, it is likely that the READ1 element in *DCDC2* and the causative variant tagged by the *KIAA0319* risk haplotype have the strongest effects on gene expression and the ultimate neurocognitive phenotype. Supporting this idea is the fact that so many of the association hits in the study—both single-marker and haplotype-based, and with all three phenotypes—localize to the same LD block as the *KIAA0319* risk haplotype. This result, together with the *KIAA0319* risk haplotype’s association with reduced *KIAA0319* expression and its synergistic interaction with a regulatory element in an intron of *DCDC2*, strongly suggests the presence of at least one regulatory variant in this region that influences *KIAA0319* expression. The locations of the only other independent hits in the locus (aside from READ1 in *DCDC2*)—an intron of *FAM65B* and downstream of a pseudogene—may suggest additional regulatory regions that influence gene expression. Thus, any roles *FAM65B* and *CMAHP* play in RD and LI may be of small effect and modulatory in nature. Though much further work is needed, we postulate, based on these and previous results, that *DCDC2* and *KIAA0319* are the major effector genes responsible for DYX2’s influence on RD and LI risk and that alteration of gene expression levels or patterns is the mechanism by which this effect is exerted.

In our study design, we emphasized replication of genetic associations in independent cohorts, as opposed to reliance on statistical corrections for multiple testing, for validation of associations in the ALSPAC discovery cohort. The replications of genetic association with our neurocognitive traits of interest, particularly in the varied cohorts in this investigation, provide strong evidence that the results of this study are not due to type I error. However, we also report uncorrected *p* values and a statistical threshold correcting for 195 genetic markers (threshold of 0.000256) to present the reader with the context of our findings in terms of multiple testing. Nonetheless, our three replication cohorts were not identical and had inherent differences to each other and relative to the discovery cohort that may have prevented replication. These differences included (1) the disorder each cohort was selected for (RD vs. LI vs. unselected), (2) severity of case definition and recruitment, and (3) country of recruitment (UK vs. US vs. Italy), and language spoken (English vs. Italian). Iowa LI and Colorado RD had moderate case definitions, which may be more comparable to Moderate RD, Moderate NWR, and Moderate WOLD case definitions in ALSPAC. On the other hand, Italy RD used a more severe case cutoff of 2 standard deviations, which may be more comparable to severe RD and severe LI case definitions in ALSPAC. Regardless, our observation of multiple replicated associations throughout the DYX2 locus increases confidence in these results.

In summary, our analyses indicate four association signals for RD, LI, and Verbal IQ in the DYX2 locus: *DCDC2*, *KIAA0319*, *FAM65B*, and the pseudogene *CMAHP*. Our association results within the *DCDC2* and *KIAA0319* (including *TDP2*, *ACOT13*, and *C6orf62*) areas are in LD with two previously reported risk variants: the READ1 regulatory element in *DCDC2* and the *KIAA0319* risk haplotype in *KIAA0319* and *TDP2*. These results point strongly, albeit circumstantially, to variation in gene expression as a mediator of DYX2’s effect on reading and language phenotypes. As these variants appear to demonstrate pleiotropy, the role of DYX2 in other neurobehavioral disorders, including attention deficit-hyperactivity disorder, autism spectrum disorders, and speech-sound disorder, awaits full characterization to determine the potentially wide range of effects the DYX2 locus has upon the brain and behavior.

## Electronic supplementary material

Below is the link to the electronic supplementary material.

Supplementary material 1 (DOCX 15 kb)
