# Supplementary material for: Characterization of the DYX2 locus on chromosome 6p22 with reading disability, language impairment, and IQ
Source: Hum Genet. 2014 Feb 9;133(7):869–81. doi: 10.1007/s00439-014-1427-3 (PMC4053598; doi:10.1007/s00439-014-1427-3)
Supplement: Supplementary file 1 — Supplementary material 1 (DOCX 15 kb) [file 439_2014_1427_MOESM1_ESM.docx]

Supplemental Table 1: Sequenom multiplex reactions

| Multiplex Reaction | N Markers |
| --- | --- |
| Plex 1 | 33 |
| Plex 2 | 33 |
| Plex 3 | 33 |
| Plex 4 | 35 |
| Plex 5 | 36 |
| Plex 6 | 34 |
| Plex 7 | 33 |
| Plex 8 | 30 |
| Plex 9 | 33 |
| **Total** | **300** |

*These reactions also contain markers outside the scope of this manuscript.
